# Sequestration of an Azo Dye by a Potential Biosorbent: Characterization of Biosorbent, Adsorption Isotherm and Adsorption Kinetic Studies

**DOI:** 10.3390/molecules29102387

**Published:** 2024-05-19

**Authors:** Bharti Gaur, Jyoti Mittal, Syed Ansar Ali Shah, Alok Mittal, Richard T. Baker

**Affiliations:** 1 Department of Chemistry, Maulana Azad National Institute of Technology, Bhopal 462 003, Indiajyalmittal@yahoo.co.in (J.M.); 2 Department of Chemistry, University of St. Andrews, Fife, St. Andrews KY16 9ST, UK; saas1@st-andrews.ac.uk

**Keywords:** hen feather, metanil yellow, adsorption isotherm, adsorption kinetics, thermodynamics

## Abstract

This study explores the detailed characterization of a biosorbent (Hen Feather) and its efficient use in eradicating the azo dye Metanil Yellow (MY) from its aqueous solutions. Effects of a range of experimental parameters, including pH, initial dye concentration, biosorbent dosage and contact time on the adsorption, were studied. A detailed physical and chemical characterization of the biosorbent was made using SEM, XRD, XPS and FTIR. During the optimization of adsorption parameters, the highest dye uptake of almost 99% was recorded at pH 2, dye concentration 2 × 10^−5^ M, 0.05 g of biosorbent and a contact period of 75 min. Various adsorption isotherm models were studied to gather different adsorption and thermodynamic parameters. The linearity of the Langmuir, Freundlich and D-R adsorption isotherms indicate homogeneous, multilayer chemisorption with high adsorption affinity between the dye and biosorbent. Values of the changes in the Gibbs free energy (ΔG°) and the enthalpy (ΔH°) of the adsorption process have been calculated, these values indicate that it is a spontaneous and endothermic process. Kinetics of the adsorption were also measured, and it was established that the adsorption of MY over Hen Feather follows a pseudo-second-order kinetic model at temperatures 30, 40 and 50 °C. The findings of this investigation clearly indicate that the studied biosorbent exhibits a high affinity towards the dye (MY), and it can be effectively, economically and efficiently used to sequestrate and eradicate MY from its aqueous solutions.

## 1. Introduction

Water contamination is a severe threat to humanity. Rapid population increase leading to speedy industrialization is the main cause of water pollution. Industrial effluents contain a variety of chemical impurities in dissolved and suspended forms, of which dissolved nonbiodegradable materials such as bulky organic dyes are considered to be highly toxic to living beings and the most difficult to remove. These organic dyes also obstruct sunlight, which impairs photosynthesis in water resources and harms aquatic life [1,2]. The removal of dyes from water via a safe method is essential, and adsorption is an established economic, effective and operationally practicable technique [3]. In adsorption operations, the selection of an adsorbent determines the efficacy and cost-effectiveness of the procedure [4]. Hence, a cheap and easily available waste material with extraordinarily high adsorption abilities can be an excellent choice as an adsorbent [5].

Previous studies claim that, of the available variety of dyes, dyes belonging to the azo group pose severe toxicity due to their carcinogenic and mutagenic behaviour [6]. The azo dye studied in this work, Metanil Yellow (MY), contains diazotized metanilic acid and diphenylamine. It is widely applied to stain paper, nylon, silk, wool and other materials and effluents of these industries contain large amounts of this toxic dye. Due to the resemblance in colour, adulterators replace costly turmeric with MY in many edible products, and this is one of the menaces of the usage of this dye [7]. Indeed, its use as a food additive has been banned due to its established severely toxic nature [8]. Many studies have confirmed that MY can lead to cardiovascular disease, damage the central venous region of liver tissue and cause histopathological abnormalities in the kidneys of goats [9]. Exposure of fish to MY results in collapsed cytoplasm, nucleus pyknosis and cardiotoxicity [10].

Based on several observations, it has been discovered that MY harms the testicles and reproductive systems of rats and guinea pigs [11,12]. It also causes inflammatory irritation when it comes into contact with the skin. It is now well established that the presence of harmful methemoglobinemia and cyanosis caused by MY adversely affects humans. Hence, when MY is ingested orally, all crucial human organs (heart, liver, kidneys, intestines, reproductive systems, neurological system, gastric tissues, etc.) can be harmed [10,13]. Therefore, keeping the toxicity of MY in view, the eradication of MY from its aqueous solution using a safe method like adsorption is highly desirable.

In the present work for the removal of MY, a bio-waste material, Hen Feather (HF), has been employed as the adsorbent since no attempt has so far been made to adsorb MY using HF. It contains a highly flexible and porous structure in the soft and the hard parts and is distinct from any other natural or artificial fibres. The shaft of the feather called the rachis is the hard part, while barbs, which originate from the rachis, and barbules, which emerge as branches from barbs, are both considered soft parts [14,15]. Chemically, Hen Feathers possess organic materials, especially protein (approximately 84%) [16]. It is pertinent to note that the soft parts of Hen Feathers (barbs/barbules) have a special cross-section that is not found in other protein fibres like wool and silk. The porous nature and high surface area of Hen Feather make it a potent biosorbent for the eradication of bulky organic dyes.

The present paper is an attempt to first explore the physical and chemical structure of the Hen Feather using a variety of analytical techniques, including scanning electron microscopy (SEM), X-ray diffraction (XRD), X-ray photoelectron spectroscopy (XPS) and Fourier transform infra-red (FTIR) spectroscopy, and then to carry out detailed and systematic studies on the adsorption of MY over Hen Feathers in aqueous solution.

## 2. Results and Discussions

### 2.1. Characterization of Biosorbent

**Figure 1:**
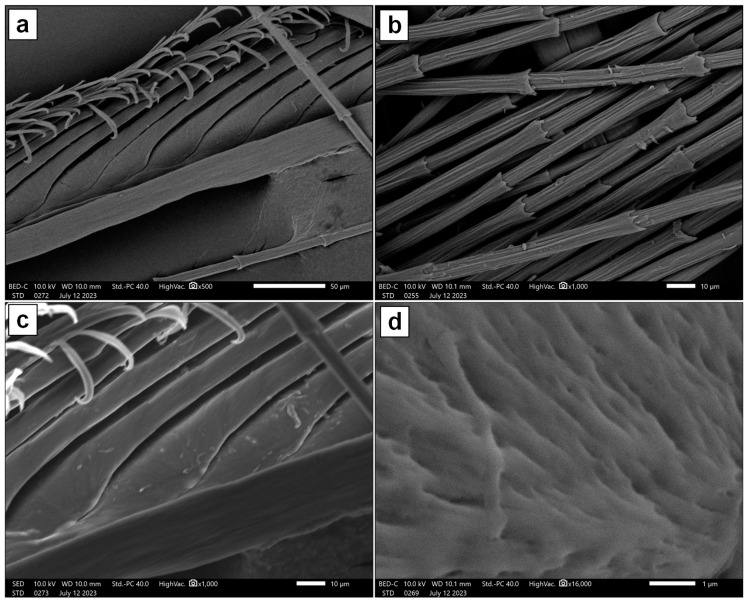
SEM images of Hen Feather taken at increasing magnifications and in both backscattered (**a**,**b**,**d**) and secondary (**c**) electron modes.

**Figure 2:**
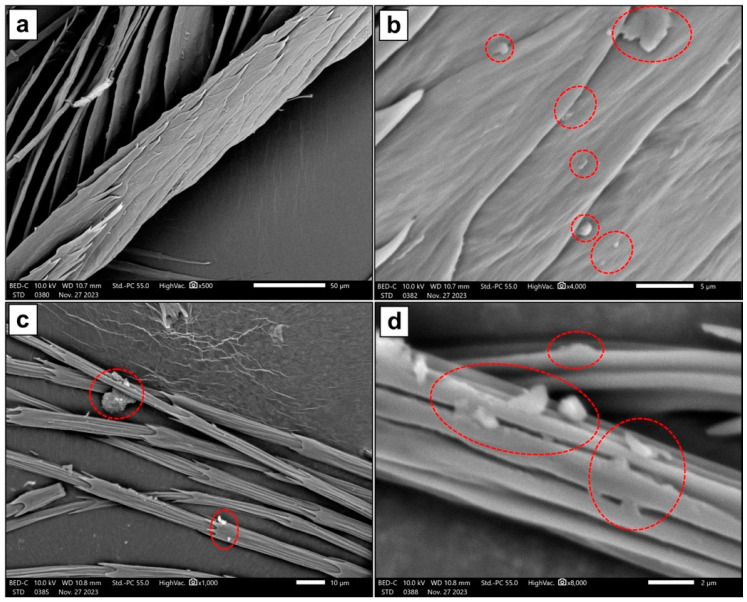
SEM images of Hen Feather treated with Metanil Yellow taken at a range of magnifications and in backscattered electron mode. Both flighted feather (**a**,**b**) and bamboo-like structures (**c**,**d**) are shown. (Examples of extraneous material, which may be aggregations of the dye, are circled in red).

The SEM images presented in Figure 1 show the microstructure of the Hen Feather sample. Figure 1a,c exhibit the branch-like ramus, which supports the barbules sprouting from it. Both distal and proximal barbules can be seen, and a series of hooks that connect these together are also visible at the top of each of these images. Bamboo-like structures are seen in Figure 1c, and the material of a single barbule is viewed at a higher magnification in Figure 1d. Figure 2 presents SEM images of the Hen Feather sample after treatment with MY solution and drying. The presence of particles and plate-like structures, which may be aggregations of MY dye molecules, indicates that MY covers both flighted and bamboo-like feathers.

**Figure 3:**
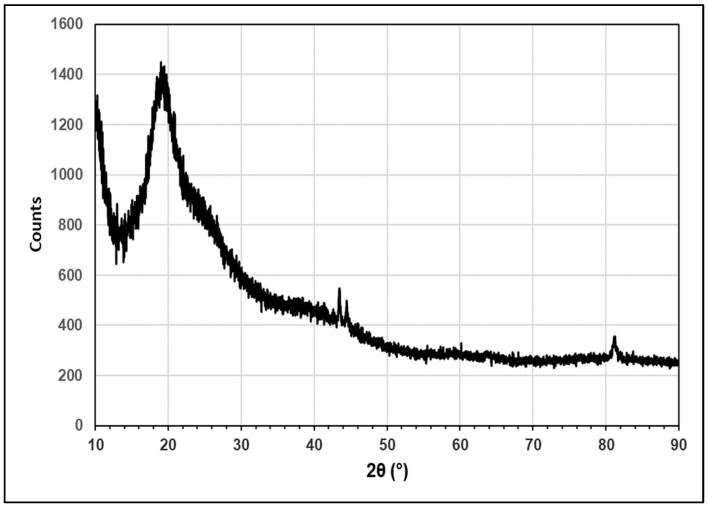
XRD pattern of pressed Hen Feather.

The XRD pattern presented in Figure 3 is very similar to patterns given for Hen Feathers and for pure keratin in the literature [17]. According to literature reports, the broad peak at ~19° results from the overlap of a peak at 17.8° and 19°, which correspond, respectively, to the α-helix and β-sheet structures [18]. A further peak at ~10°, which can be seen in Figure 3, also corresponds to the α-helix structure [19]. Since both broad peaks are intense, it is clear that both these structural conformations are common in this sample.

**Figure 4:**
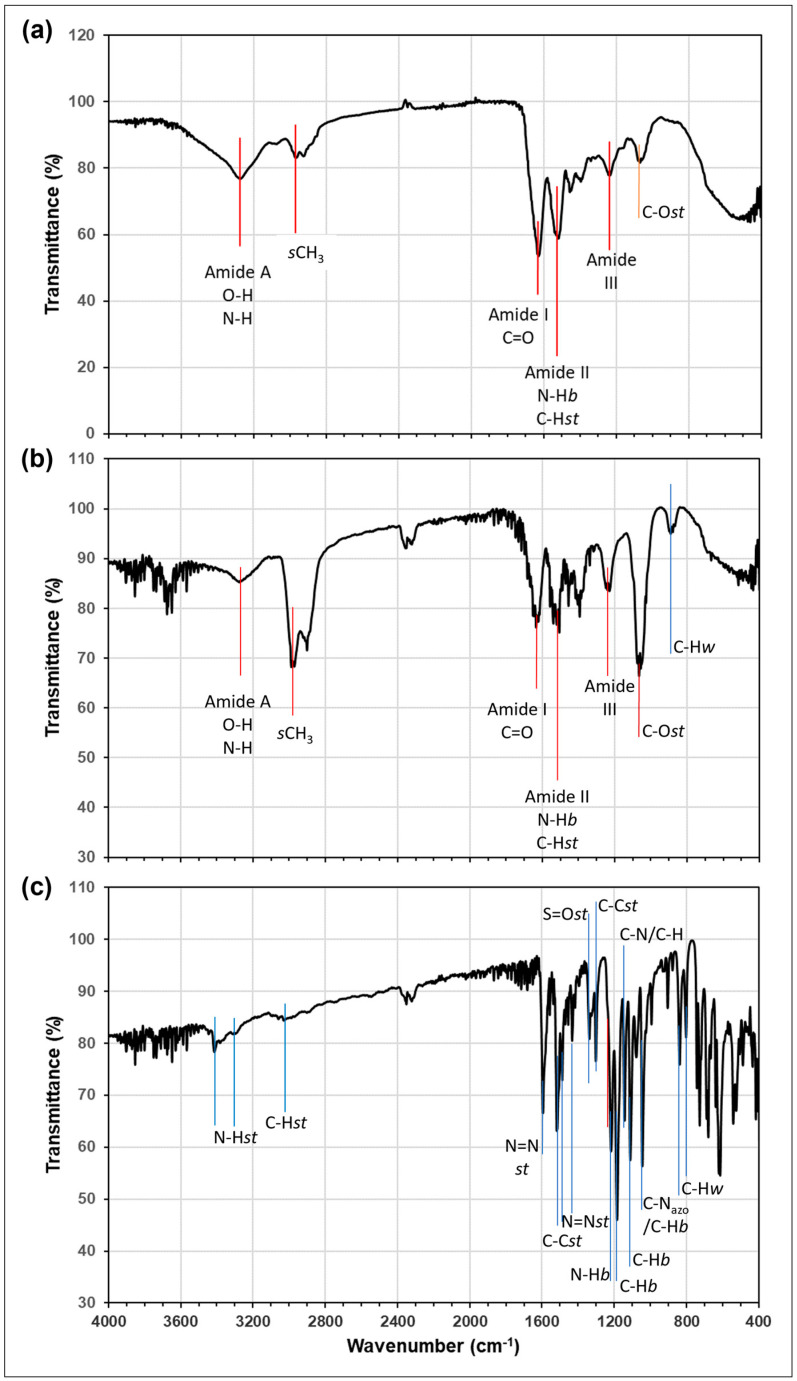
Fourier transform infra-red spectrum of (**a**) Hen Feather. (**b**) Hen Feather treated with MY solution (pH 2.0) and dried and (**c**) pure crystalline MY (*s*CH_3_: symmetric mode of CH_3_ Ion, *b*: bend and *st*: stretch).

The FTIR spectrum of the Hen Feather is presented in Figure 4a. Previous studies [17] have shown that the FTIR spectra of Hen Feather and of pure keratin are very similar, indicating a high content of the latter in the former. The spectrum presented here is consistent with this. The main peaks are reported to relate to the peptide bonds in the keratin (-CONH) and have been labelled according to the convention as Amides A, I, II and III. The band at ~3270 cm^−1^ can be due to stretching vibrations of O–H and N–H and is known as Amide A. The band is at 1630 cm^−1^ (labelled as Amide I) and relates to a C=O stretch. The band at 1520 cm^−1^ (Amide II) is due to a C–H stretch and N–H bend, while the band at ~1230 cm^−1^ is a combination of several vibrations (C=O bend, C–C stretch, N–H bend and C–N stretch) and is labelled Amide III. Amide A is reported to relate to the α-helix structure of keratin; Amide II to the β-sheet structure; and Amide III to the combination of both of these structures. On this basis, both structures must be present in this Hen Feather sample. The spectra of Hen Feather after treatment with MY, and of pure crystalline MY are present in Figure 4b,c, respectively. The major vibrational bands assigned for MY by Dhakal et al. [7] are labelled on Figure 4c. Notable among these are the N-H stretch (3412, 3294 cm^−1^), aromatic C-H stretch (~3020 cm^−1^), stretching modes of the azo group, N=N, (1595, 1435 cm^−1^), the stretch of the neighbouring C-N_azo_ bonds (1045 cm^−1^) and the S=O stretch (1339 cm^−1^) of the sulfonic acid group on MY. In the MY-treated Hen Feather material, the Amide bands for the O-H/N-H, C=O and C-O bonds appear at the same frequencies as for the untreated sample. This suggests that the extensive hydrogen bonding present between carbonyl and the N atom in the keratin, which is a major component of Hen Feather, is not significantly changed by the addition of the dye. However, it should be noted that the relative intensity of the C-O peak at 1066 cm^−1^ does increase significantly. The dye was added to the Hen Feather by soaking it in an acidic solution of MY (pH 2), followed by drying. This may cause protonation of a proportion of the amide carbonyl species, giving a larger concentration in the enol form [20], resulting in a higher concentration of singly bonded C-O than before treatment with the MY solution.

**Figure 5:**
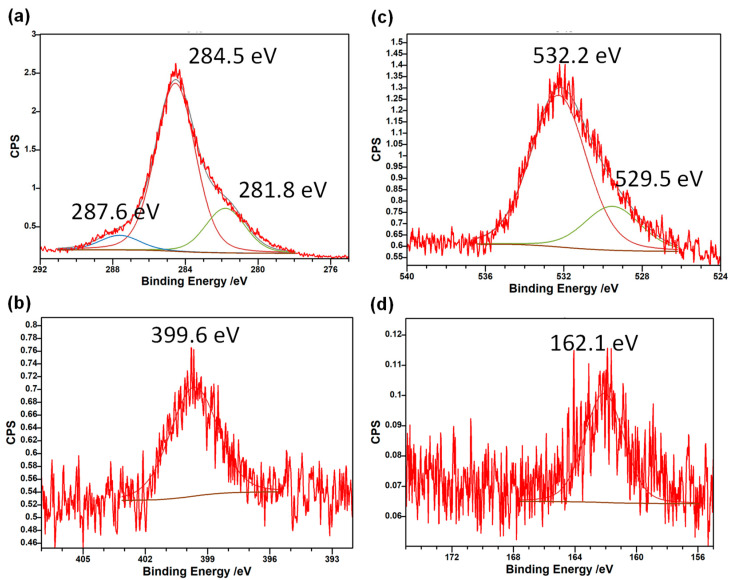
X-ray photoelectron spectra of Hen Feather at (**a**) C 1s, (**b**) N 1s, (**c**) O 1s and (**d**) S 2p regions.

XPS spectra of the Hen Feather sample are presented in Figure 5. The atomic surface composition of the sample was determined to be 85.4% C, 11.1% O, 2.9% N and 0.72% S. This is consistent with Hen Feather consisting predominantly of the polypeptide; keratin, which contains –CONH– linkages between amino acid units; and –S–S cross-linking. No charging of the sample was evident. The main C 1s peak at 284.5 eV is consistent with C–C and C–H environments. The small peak at 287.6 eV would match C in C=O groups, while another small peak at low binding energy—281.8 eV—is an artifact due to the use of charge compensation in the XPS instrument and can be ignored. The single peak for N 1s at 399.6 eV can be confidently attributed to N in amide groups, –N(C=O)–C–, which are very common in keratin. The main O 1s peak is consistent with C=O groups, while the smaller peak at 529.5 eV matches the C-O environment, which is present in some amino acids. In the S 2p region, the single peak seen should be attributed to electron-rich S environments such as S^2−^ or S_2_^2−^ groups. This agrees with the known presence of –S–S– cross-linkages in keratin and Hen Feather.

Standard chemical methods [21] were applied to analyze the Hen Feather, and the sample was found to contain a maximum of about 82% of protein, while other components like fat, ash, crude fibre, available Lysine, Methionine and Cysteine each were almost less than 2%. The approximate results of the ultimate analysis of Hen Feathers are carbon (64.5%), nitrogen (10.5%), oxygen (22%) and sulphur (3%). The porosity (74%) density (0.3834 g∙m^−3^) and surface area (1170.6 cm^2^∙g^−1^) of the Hen Feathers were determined by standard methods.

### 2.2. Preliminary Adsorption Studies

#### 2.2.1. Influence of pH

**Figure 6:**
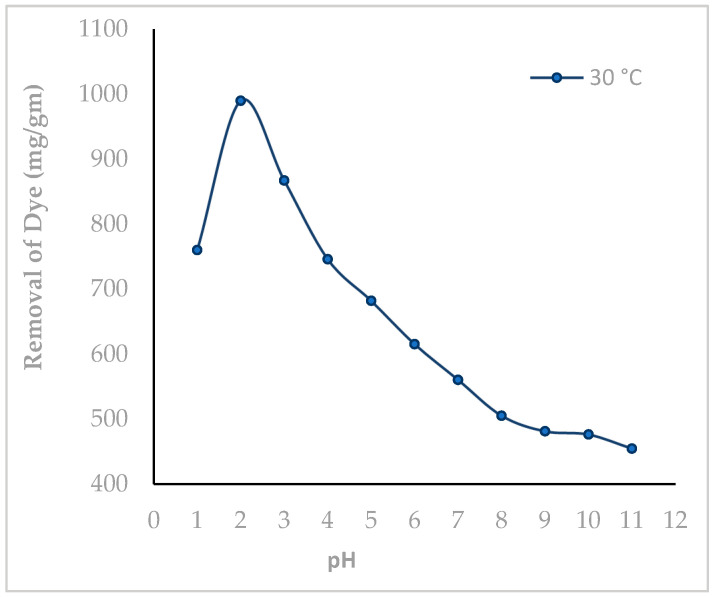
Influence of pH on the adsorptive removal of the dye Metanil Yellow over Hen Feathers (initial dye concentration: 2 × 10^−5^ M, adsorbent dosage: 0.05 g/20 mL, contact time: 75 min).

To measure the effect of pH on the dye removal, 2 × 10^−5^ M MY was taken in 10 different flasks. The pH of each flask was maintained from 1.0 to 11.0. Figure 6 indicates that the highest dye uptake of about 99% was obtained at pH 2.0, and with increasing pH dye uptake, decreases almost linearly to about 45% at pH 11.0. Since the highest adsorption is achieved at pH 2.0, this pH was selected to carry out all subsequent studies.

In the strong acidic medium at pH 2.0, electrostatic attraction of deprotonated MY and protonated HF results in the strong adsorption of MY over HF. With increasing pH, weaker electrostatic attraction force develops, which reduces the dye removal [22,23].

In order to determine the nature of the Hen Feathers, its weighed quantities (0.05, 0.10, 0.20, 0.30, 0.40 and 0.50 g) were dipped in 25 mL of distilled water (pH = 7.0), and the mixtures were taken in six 100 mL airtight measuring flasks. After almost 24 h, each mixture was filtered, and the pHs of the filtrates were recorded. It is interesting to note that each sample exhibited an increase in pH, thereby indicating the basic nature of Hen Feathers.

#### 2.2.2. Influence of Biosorbent Dosage

**Figure 7:**
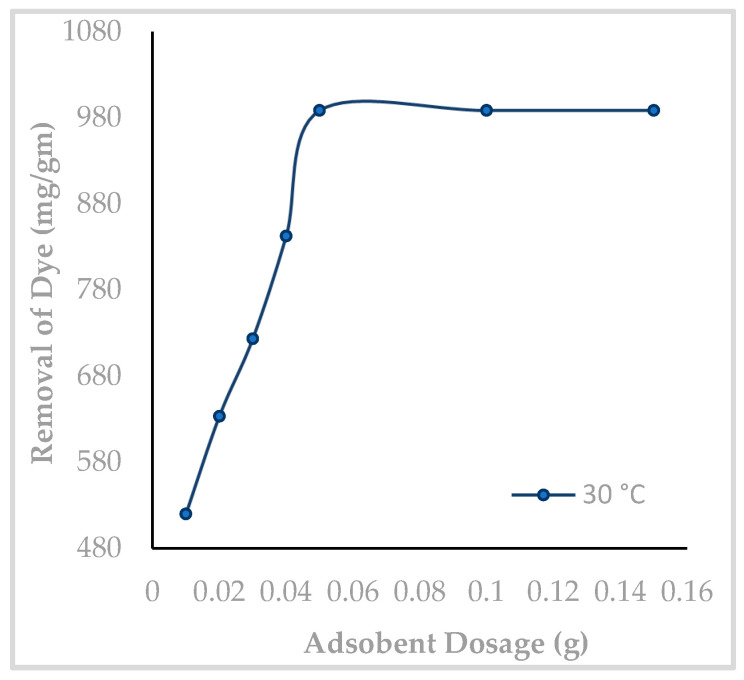
Influence of Hen Feather dosage on the adsorptive removal of Metanil Yellow (pH: 2, initial dye concentration: 2 × 10^−5^ M, contact time: 75 min).

Adsorption studies were performed by adding 0.01 to 0.15 g of Hen Feathers to a dye solution of concentration 2 × 10^−5^ M and pH 2. After agitating the solution for 75 min, the uptake of MY was monitored, and the results obtained are presented in Figure 7. It is found that initially, the percentage removal of MY increases with increasing amounts of Hen Feathers in the solution, and the highest dye uptake was recorded at a biosorbent amount of 0.05 g. Beyond this value, the dye uptake was constant. An increase in the dye adsorption is due to the availability of large numbers of binding sites; 0.05 g HF may be the reason for the increased adsorption of MY.

#### 2.2.3. Influence of Adsorbate Concentration

The effect of MY concentration on its removal at different temperatures was observed at pH 2.0 and by adding 0.05 g of Hen Feathers. At each temperature, the percentage removal increased linearly and attained a plateau at 2 × 10^−5^ M dye concentration. The increase in the amount of the MY with an increase in concentration may be due to large numbers of available binding sites of the biosorbent, but at dye concentrations around 2 × 10^−5^ M and above, the binding sites become almost saturated. Thus, the percentage removal of the dye attains almost a fixed value at each temperature.

#### 2.2.4. Influence of Contact Time

**Figure 8:**
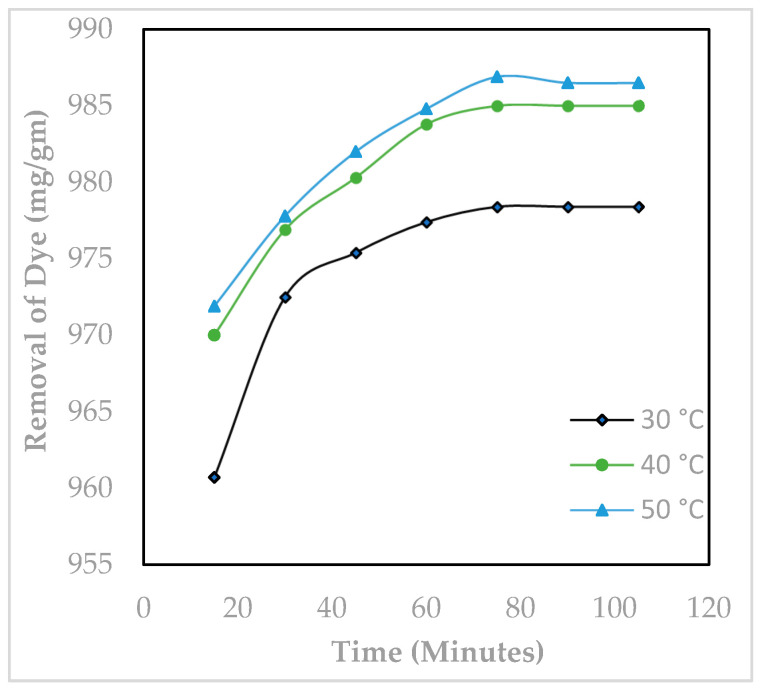
Influence of contact time on the adsorptive removal of Metanil Yellow over Hen Feathers (pH: 2.0, initial dye concentration: 2 × 10^−5^ M, adsorbent dosage: 0.05 g/20 mL).

To measure the effect of time of contact of MY and Hen Feather, their solutions were thoroughly agitated at different time periods (Figure 8). Figure 8 shows that 75 min are sufficient to attain equilibrium of adsorption of the MY–Hen Feather. Figure 8 clearly indicates that the proportion of MY adsorption increases steadily from a contact time of 15 to 75 min and then starts stabilizing due to coverage of the Hen Feather by MY.

### 2.3. Adsorption Isotherm Studies

Adsorption isotherms provide a great deal of information on the interaction in the adsorbate–adsorbent system, particularly adsorption behaviour, binding energy, thermodynamic parameters and nature of adsorption. Here, Langmuir, Freundlich, Temkin and Dubinin–Radushkevtich adsorption isotherms were all examined. The theory and other details regarding each adsorption isotherm are well documented in the literature [24,25]. In each case, experiments were performed by varying dye concentration and temperature and keeping the optimum values of other parameters as determined in the previous section.

#### 2.3.1. Langmuir Adsorption Isotherm

It is well known that in the year 1916, Irving Langmuir postulated an experimental isotherm model. The model is helpful in establishing monolayer adsorption over homogeneous surfaces and in providing values of different thermodynamic parameters like change in Gibb’s free energy (ΔG°), enthalpy (ΔH°), entropy (ΔS°), etc., during the adsorption process. The linear form of the Langmuir adsorption isotherm model can be expressed as

(1) $$\frac{1}{q_{e}}=\frac{1}{q_{o}}+\frac{1}{bq_{o}c_{e}}$$

**Figure 9:**
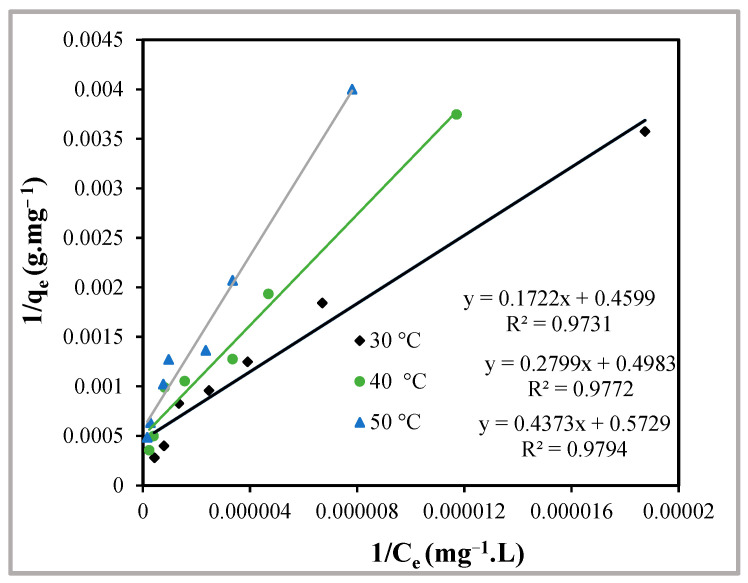
Langmuir adsorption isotherms at the temperatures indicated for the adsorption of Metanil Yellow over Hen Feathers at pH = 2.

The amount of adsorbate adsorbed at equilibrium (mg∙g^−1^) is denoted by *q*_e_, *C*_e_ denotes the dye’s equilibrium molar concentration (mg∙L^−1^), q_o_ is the adsorbent’s maximum adsorption capacity per unit mass (mg∙g^−1^) and b is the Langmuir constant (L∙mg^−1^). Graphs of 1/*C*_e_ versus 1/*q*_e_, plotted for different temperatures, give straight lines with regression coefficients close to unity (Figure 9). This indicates that the data obtained follow the Langmuir isotherm model, and the monolayer adsorption of the dye, MY, takes place over the homogeneous surface of Hen Feathers at all temperatures studied. The values of Langmuir adsorption constant ‘*b*’ are given in Table 1 and decreases with increase in temperature. High values of ‘*b*’ indicate strong interaction between MY and Hen Feather. However, with increasing temperatures, the value of ‘*b*’ decreases, thereby indicating weaker adsorption at higher temperatures.

The values of ‘*b*’ were applied to evaluate a dimensionless separation factor (*r*), which is helpful in establishing the favourability of the ongoing adsorption process. The values of ‘*r*’ can be evaluated using the following equation:

(2) $$r=\frac{1}{1+bC_{o}}$$

Here, *b* and *C_o_* have the same meanings as described above. At temperatures 30, 40 and 50 °C, the values of ‘*r*’ are found to be 0.047, 0.069 and 0.092. Since these values all fall between 0 and 1, the adsorption of MY over Hen Feathers can be considered a favourable process.

#### 2.3.2. Freundlich Adsorption Isotherm

The Freundlich adsorption isotherm is related to multilayer formation over a heterogeneous surface and is mathematically expressed by the following expression:

(3) $$logq_{e}=logk_{f}+\left(\frac{1}{n}\right)logC_{e}$$

Here, k_f_ and n are the Freundlich constants, determined by the intercept and slope, respectively, C_e_ (mol∙L^−1^) is the equilibrium concentration and q_e_ (mol∙g^−1^) is the equilibrium capacity of the adsorbent.

**Figure 10:**
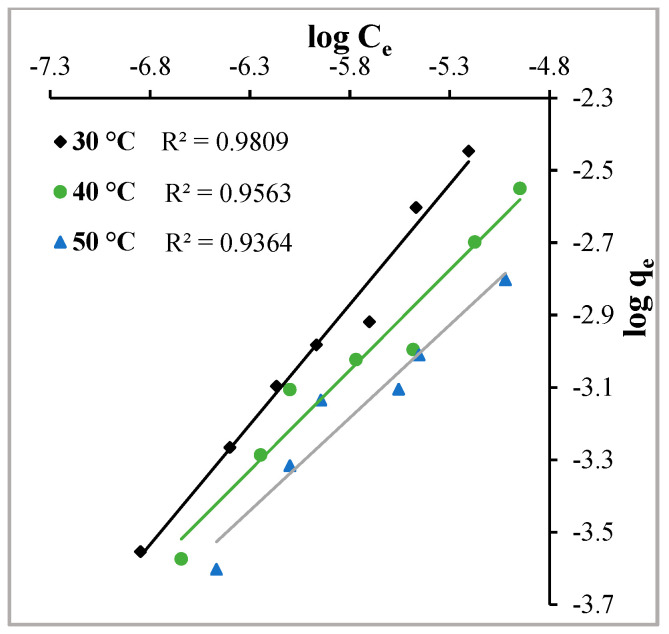
Freundlich adsorption isotherms at the temperatures indicated for the adsorption of Metanil Yellow over Hen Feather at pH = 2.

To verify the applicability of the Freundlich adsorption isotherm model, graphs of log C_e_ versus log q_e_ were plotted (Figure 10). Figure 10 exhibits that straight lines with negative intercepts and regression coefficients close to unity were obtained at all temperatures. This indicates the adsorption of the dye MY over Hen Feathers can follow a multilayer formation even at low concentrations. Table 1 presents the values of various Freundlich constants.

#### 2.3.3. Dubinin–Radushkevtich Adsorption Isotherm

**Figure 11:**
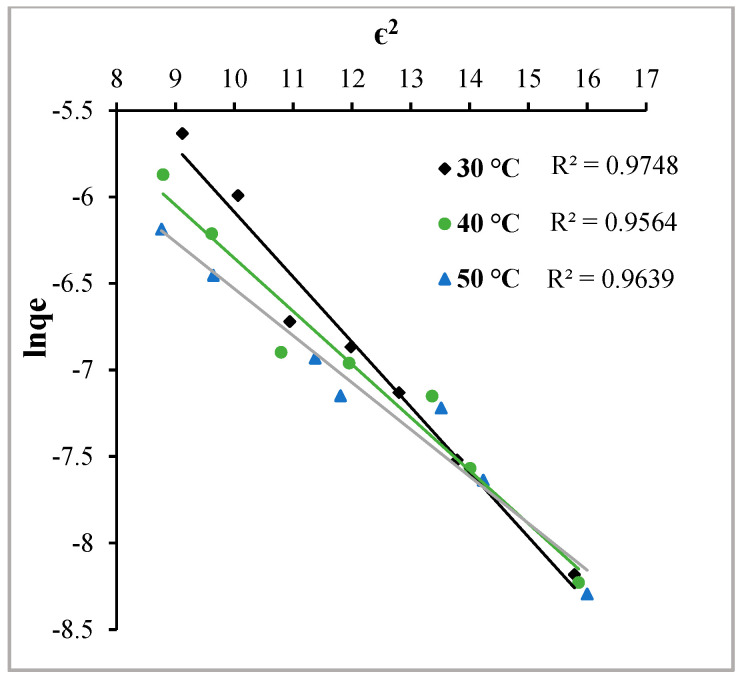
Dubinin–Radushkevitch adsorption isotherms at the temperatures indicated for the adsorption of Metanil Yellow over Hen Feathers at pH = 2.

The Dubinin–Radushkevtich (DR) adsorption isotherm model is helpful in diagnosing adsorption behaviour, specifically whether the adsorbing molecules are undergoing chemisorption or physisorption. The following is the linear form of the DR adsorption isotherm model:

(4) $$ln\;C_{ads}=ln\;X_{m}-\beta \in^{2}$$

where C_ads_ (mol∙g^−1^), X_m_ (mol∙g^−1^) and β (mol^2^∙J^−2^) are the amount of MY adsorbed per unit weight of Hen Feathers, the maximum adsorption capacity of Hen Feathers and the activity coefficient related to the mean adsorption energy, respectively. The Polanyi potential (∈) is given as

(5) $$\in =RT\cdot ln\left(1+\frac{1}{C_{e}}\right)$$

where T is the Kelvin temperature, and R (8.314 J∙mol^−1^K^−1^) is the Gas Constant. The graph of ∈^2^ vs. lnC_ads_ is presented in Figure 11. The straight lines depict regression coefficient values of near unity at all temperatures, indicating that the DR adsorption model can be applied in the ongoing adsorption process (Table 1). To ascertain whether the process is physisorption or chemisorption, mean sorption energy (E) was calculated using the following relationship:

(6) $$E=\frac{1}{\sqrt{-2\beta }}$$

Hutson and Yang [26] determined that for any adsorbate–adsorbent system, if the value of ‘E’ is less than 8 kJ∙mol^−1^, physisorption dominates, while a value between 8 and 16 kJ∙mol^−1^ ascertains the chemisorption process. Due to values of more than 8 kJ/mol at all temperatures, it can be safely interpreted that the ongoing adsorption is chemisorption only.

#### 2.3.4. Thermodynamic Parameters

The measurement of thermodynamic parameters is crucial for determining the process’s viability and feasibility as well as for understanding the effect of temperature on MY adsorption. Using Equations (7) to (9), values of ΔG°, ΔH° and ΔS° associated with the ongoing adsorption process were calculated (Table 2).

(7) $$∆G^{\circ }=-RTlnb$$

(8) $$∆H^{\circ }=-R\left(\frac{T_{2}T_{1}}{T2-T1}\right)\times ln\left(\frac{b_{2}}{b_{1}}\right)$$

(9) $$∆S^{\circ }=\frac{∆H^{\circ }-∆G^{\circ }}{T}$$

The negative values of ∆G° indicate that the process of adsorption of MY on Hen Feathers is feasible, while the positive values of ΔH confirm the endothermic nature of the adsorption. Similarly, by using Equation (9), it is ascertained that entropy (∆S°) is positive, thereby indicating an increased randomness at the MY–Hen Feather interface with minor structural changes in the Hen Feather.

### 2.4. Kinetic Studies

In the kinetic studies, the rate and order of the adsorption process are calculated by monitoring the effect of contact time on the percentage removal of the dye. An amount of 0.05 g of Hen Feathers was added to a dye solution of 2 × 10^−5^ mole∙L^−1^ concentration and pH 2.0 at fixed temperatures (30, 40 and 50 °C), and the flask was agitated on a mechanical shaker for a predetermined amount of time. To calculate the order of reaction, two well-established rate equations, namely Legergren’s rate equation (Equation (10)) and the Ho–McKay rate equation (Equation (11)) for pseudo-first-order and pseudo-second-order reactions, respectively, were applied [27,28].

(10) $$log(q_{e}-q_{t})=logq_{e}-\frac{k_{1}}{2.303}\times t$$

(11) $$\frac{t}{q_{t}}=\frac{1}{k_{2}{q_{e}}^{2}}+\frac{t}{q_{e}}$$

Here, q_e_ (mol∙g^−1^), q_t_ (mol∙g^−1^), k_1_ (min^−1^) and k_2_ (g∙mol^−1^.min^−1^) are the amount adsorbed at equilibrium, amount adsorbed at time ‘t’, pseudo-first-order rate constant and pseudo-second-order rate constant, respectively.

**Figure 12:**
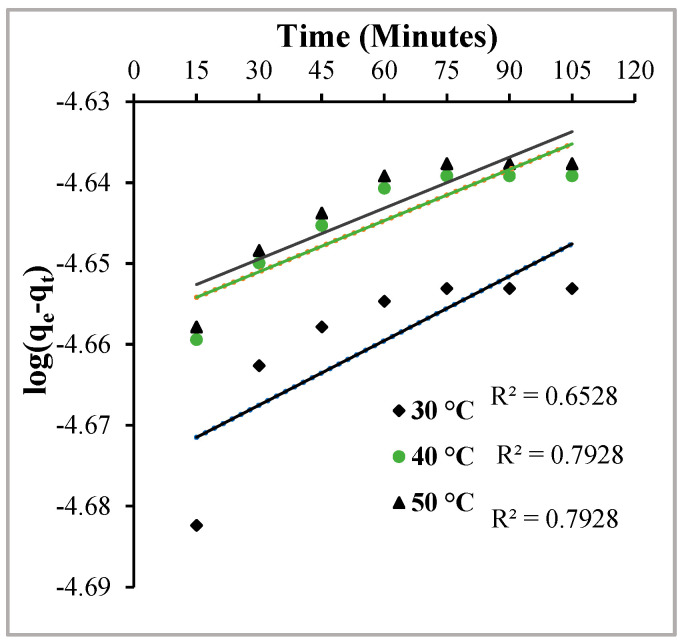
Legergren’s plot for the adsorption of Metanil Yellow over Hen Feathers at the indicated temperatures.

**Figure 13:**
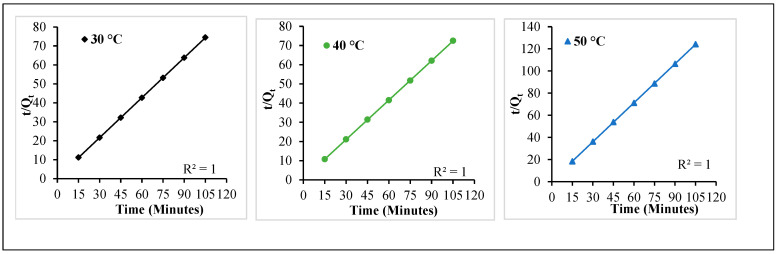
Ho and McKay’s plot for the adsorption of Metanil Yellow over Hen Feathers at the indicated temperatures.

To establish an order of ongoing adsorption, using Equation (10), a plot of time vs. log(q_e_ − q_t_) and using Equation (11), a graph of time versus t/q_t_ was plotted (Figure 12 and Figure 13). It is clear from Figure 13 that the values of the regression coefficients of the straight lines obtained at all three temperatures are close to unity, while in Figure 12, the lines obtained have low regression coefficient values. Thus, it can be safely interpreted that pseudo-second-order rate kinetics are operative in the MY adsorption over Hen Feather, and the rate constants of the reaction (k_2_) are 2.933 × 10^4^, 1.40 × 10^3^ and 2.41 × 10^3^ (L∙mol^−1^ s^−1^) at 30, 40 and 50 °C, respectively.

### 2.5. Mechanism of MY Adsorption over Hen Feathers

With the results mentioned above, it can be determined that the azo dye MY adsorbs over Hen Feathers via chemisorption. First, a uniform monolayer is formed, which can extend to form multilayers. The FTIR spectrum of MY-treated Hen Feather in Figure 5 confirms the presence of N=N, N–H and S=O bonds. In acidic media, the azo bond would be expected to be protonated to give the –HN^+^=N– bond. On the other hand, FT-IR studies of the Hen Feather indicate the presence of amide linkages containing C=O and N–H bonds, and these are known to hydrogen bond to form keratin sheets in feathers. The adsorption isotherm studies and evaluated thermodynamic parameters clearly indicate that the adsorption of MY over Hen Feathers undergoes a spontaneous chemisorption process with strong interactions. Therefore, it can be determined that chemical bond formation (chemisorption) will take place between the N–H, azo groups and the sulfonyl species of the MY molecule and the C=O and N-R groups of the Hen Feathers. The presence of these bonds on the MY molecules and keratin structures would be expected to give rise to strong bond formation.

## 3. Experimental

### 3.1. Material and Methods

**Figure 14:**
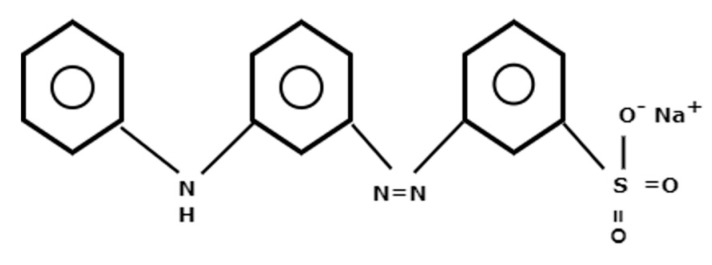
Chemical structure of Metanil Yellow.

MY (Figure 14) is an azo dye containing the –N=N– group. The IUPAC name of MY (C_8_H_15_N_3_NaO_3_S) is sodium 3–[4anilinophenyl)diazinyl]benzene sulfonate. It is a yellow water-soluble dye with a molecular weight of 376.39 and a melting point of >250 °C. AR-grade MY was procured from M/s Merck. All working solutions of MY were prepared in double-distilled water after diluting its 1 M stock solution. The pH of the working solutions was adjusted using HCl and NaOH.

The biosorbent Hen Feathers were obtained from local poultry farmers. The feathers were washed and activated before use. The characterization of Hen Feathers was carried out using a range of techniques. SEM photographs were obtained using a JEOL JSM-IT200 instrument (JEOL UK Ltd., Welwyn Garden City, UK) operating at 10 kV at a working distance of 10 mm in both secondary electron and backscattered electron modes. XRD patterns of Hen Feathers were collected on a PANalytical Empyrean instrument (Malvern Panalytical, Malvern, UK) using Cu K_a1_ radiation (1.5406 Å) in reflection mode over a 2θ range of 10–90°. XPS was carried out on a Scienta XPS spectrometer (Scienta Omicron, Uppsala, Sweden) with a monochromatic source. Using a Shimadzu IRAffinity 1S spectrometer (Kyoto, Japan), FTIR spectra were obtained over the range of 400–4000 cm^−1^.

To carry out all adsorption studies, a water bath shaker (RSB-12) of M/s Remi (Mumbai, India) a microprocessor-based pH system (model no. 1013) of M/s ESICO (Parwanoo, India) and a double-beam spectrophotometer (M/s ESICO India) were used.

### 3.2. Development of Biosorbent

Hen Feathers were obtained from local poultry. These were dirty and stained in blood. Therefore, these were first cleaned with water and detergent several times. Using double-distilled water, the washed material was further rinsed. In order to remove adhering organic impurities, the feathers were kept in H_2_O_2_ solution for about 24 h. The material was once again submerged in double-distilled water overnight. The Hen Feathers thus obtained were dried in a hot air oven at 80 °C. Using a pair of sharp scissors, the barbs and barbules of the Hen Feathers were first separated from the shaft and cut down to very small pieces typically 1 mm in length. The material thus obtained was stored in a desiccator.

A three-step batch adsorption study was performed, which included preliminary investigations, adsorption isotherm studies and kinetic studies. Firstly, to assess the optimum values of the parameters pH, biosorbent dosage, adsorbate concentration and contact time to remove MY from its aqueous solutions, preliminary investigations were carried out. Then, adsorption isotherm studies were carried out in a wide range of dye concentrations with appropriate amounts of Hen Feather at three different temperatures (30, 40 and 50 °C) and recording uptake of the dye. To evaluate kinetic parameters, the adsorption was monitored at different time intervals.

In each batch experiment, 20 mL of the known concentration and pH of MY was taken in a well-stoppered 100 mL flask and mixed thoroughly with the chosen amount of biosorbent (Hen Feather) at a constant temperature (30, 40 and 50 °C) and shaking speed (150 rpm) for a specific time interval. The solution was then filtered, and its absorbance was recorded at a fixed wavelength (λ_max_ = 425 nm) using the ultraviolet–visible spectrophotometer to evaluate the percentage removal of the dye.

## 4. Conclusions

The present paper focuses on the efficacy of Hen Feathers, a waste material, which is easily available in very large quantities and can be used as an adsorbent in aqueous media without compromising the cleanliness of the water. Hen Feathers are a type of biosorbent that can be successfully and directly used without any structural alteration and exhibit effective removal of dyes. The data provided in this paper demonstrate how effective Hen Feathers are at absorbing the dye MY. Furthermore, the interpretation of these data by reference to a range of appropriate adsorption models provides detailed information relevant to evaluating the practicality, cost-effectiveness and environmental friendliness of Hen Feathers for the adsorption of MY. The best adsorption of the dye can be achieved at pH 2.0. As a result, it can be safely stated that Hen Feathers are an effective and cheap waste material for the eradication of MY from its aqueous solutions.

## Figures and Tables

**Table 1:** Values of various isotherm constants for the uptake of Metanil Yellow by Hen Feathers at the indicated temperatures.

Langmuir Adsorption Isotherm			
Parameter	Temperature (°C)		
	30	40	50
q_o_ × 10^3^ (mg∙g^−1^)	2.17	2.01	1.75
b (L∙mg^−1^)	2.67	1.78	1.31
R^2^	0.9731	0.9772	0.9794
**Freundlich Adsorption Isotherm**			
K_f_ (mol∙g^−1^)	0.10	0.70	0.62
n	1.51	1.81	1.95
R^2^	0.9809	0.9563	0.9364
**Dubinin–Radushkevtich Adsorption Isotherm**			
X_m_ × 10^−2^ (mol∙g^−1^)	9.74	3.74	2.19
b × 10^−9^ (L∙mol^−1^)	4.00	3.00	3.00
E (kJ mol^−1^)	11.18	12.91	12.91
R^2^	0.9748	0.9564	0.9639

**Table 2:** Values of various thermodynamic parameters for the uptake of the dye Metanil Yellow by Hen Feathers at the indicated temperatures.

Parameter	Temperature (°C)		
	30	40	50
–ΔG° (kJ·mol^−1^)	2.47	1.50	0.73
ΔH° (kJ·mol^−1^)	31.98	25.78	12.48
ΔS° (J·K^−1^mol^−1^)	113.71	90.03	43.57

## Data Availability

Data are contained within the article.
